# Optimizing Irrigation Strategies to Improve Yield and Water Use Efficiency of Drip-Irrigated Maize in Southern Xinjiang

**DOI:** 10.3390/plants13243492

**Published:** 2024-12-13

**Authors:** Qingyong Bian, Zhiduo Dong, Yanbo Fu, Yupeng Zhao, Yaozu Feng, Zhiguo Wang, Jingquan Zhu

**Affiliations:** 1 Institute of Soil Fertilizer, Agricultural Water Saving, Xinjiang Academy of Agricultural Sciences, Urumqi 830091, China; 18116873795@126.com (Q.B.); dzd1281228561@163.com (Z.D.); 320223547@xjau.edu.cn (Y.Z.); guotaiminan7@163.com (Y.F.); wangzhiguo@xaas.ac.cn (Z.W.); 2 National Soil Quality Aksu Observation Experimental Station, Aksu 843000, China; ayg13779155706@163.com; 3 Scientific and Technological Achievement Transformation Center, Xinjiang Academy of Agricultural Sciences, Urumqi 830091, China

**Keywords:** maize, irrigation, crop coefficient, yield, irrigation water use efficiency

## Abstract

The contradiction between increased irrigation demand and water scarcity in arid regions has become more acute for crops as a result of global climate change. This highlights the urgent need to improve crop water use efficiency. In this study, four irrigation volumes were established for drip-irrigated maize under plastic mulch: 2145 m^3^ ha^−1^ (W1), 2685 m^3^ ha^−1^ (W2), 3360 m^3^ ha^−1^ (W3), and 4200 m^3^ ha^−1^ (W4). The effects of these volumes on soil moisture, maize growth, water consumption, crop coefficients, and yield were analyzed. The results showed that increasing the irrigation volume led to a 2.86% to 8.71% increase in soil moisture content, a 24.56% to 47.41% increase in water consumption, and a 3.43% to 35% increase in the crop coefficient. Maize plant height increased by 16.34% to 42.38%, ear height by 16.85% to 51.01%, ear length by 2.43% to 28.13%, and yield by 16.96% to 39.24%. Additionally, soil temperature was reduced by 1.67% to 5.67%, and the maize bald tip length decreased by 6.62% to 48%. The irrigation water use efficiency improved by 6.57% to 28.89%. A comprehensive evaluation using the TOPSIS method demonstrated that 3360 m^3^ ha^−1^ of irrigation water was an effective irrigation strategy for increasing maize yield under drip irrigation with plastic mulch in the southern border area. Compared to 4200 m^3^ ha^−1^, this strategy saved 840 m^3^ ha^−1^ of irrigation water, increased the irrigation water use efficiency by 23.96%, and resulted in only a 0.84% decrease in yield. The findings of this study provide a theoretical foundation for optimizing production benefits in the context of limited water resources.

## 1. Introduction

The importance of irrigation in ensuring food security has been well documented [1]. It has been estimated that between 70% and 90% of the world’s freshwater resources were used for agricultural production [2,3,4]. However, the challenges of water scarcity driven by climate change, population growth, and agricultural activities have become critical issues in many parts of the world, particularly in arid and semi-arid climates [5]. At the same time, the area of irrigated farmland has been projected to continue expanding in tandem with population growth and increasing food demand [5,6], which would undoubtedly intensify the pressure on agricultural water use. In the face of escalating water scarcity and the uncertainties posed by climate change, the judicious use of limited water resources to achieve sustainable increases in crop yields has become a pivotal aspect of ensuring food security [7]. The deployment of advanced irrigation technology and the formulation of optimal irrigation strategies has facilitated the rational utilization of available water resources, thereby improving overall irrigation management [8]. Studies show that ineffective water consumption in farmlands can be reduced, and soil water retention can be increased by using methods such as film covering [9], straw covering [10], subsurface drip irrigation [11], deficit irrigation [12], and applying anti-transpirant agents [13]. These practices can subsequently improve the efficiency of agricultural water use and enhance productivity [14].

Maize (*Zea mays* L.) is a significant food crop and the most consumed cereal crop globally [15,16,17]. It has the second highest total production and productivity in the world, with 260 million tons total and 6.3 tons per hectare, respectively [18]. It has been anticipated that crop production will need to increase by over 70% by 2050 to meet the food demands of the growing global population [19]. However, the future growth of maize production through an expansion in planted area is limited due to the reduction in arable land and natural resources. Consequently, increasing maize productivity per unit area has become more important, and irrigation management plays a significant role [20,21]. Therefore, improving irrigation methods represents a potential solution for achieving higher yield levels in water-scarce areas while enhancing the resilience of cropping systems to climate change [22,23].

Maize is a crop with high water consumption, and identifying optimal agronomic practices is crucial for maximizing yields while conserving water and efficiently utilizing the limited irrigation water available [24]. To alleviate water scarcity and improve irrigation water use efficiency, the adoption of mulch film drip irrigation technology represents a viable solution [25]. Mulch drip irrigation integrates advanced drip irrigation technology with cover cropping technology [26], a strategy that has been demonstrated to be an effective means of conserving water, regulating salt levels, maintaining soil moisture, enhancing crop yields, and improving water use efficiency [27]. Previous research demonstrated that maize is highly susceptible to irrigation deficiencies, which was attributed to its elevated water consumption [12], increased plant density, and other changes in crop management practices [28]. Over-irrigation was shown to reduce crop yield and irrigation water productivity, whereas deficit irrigation increased yield and reduced field crop evapotranspiration [29,30]. The latter was a significant factor in determining irrigation intensity, given that crop transpiration represents a major pathway for on-farm water depletion [31,32]. Furthermore, crop evapotranspirational water consumption is a crucial consideration in this context. As a pivotal point in the transformation of water movement and energy flux within the soil–plant–atmosphere continuum, this process integrates the physics of water movement and plant physiology. It is influenced by a range of factors, including soil moisture status, meteorological conditions, living environment, and the plant’s own growth and development [33].

The unique climatic conditions of Xinjiang support maize growth but are limited by water scarcity and soil salinization. To ensure food security, it is essential to reduce irrigation water use while maintaining crop yield. This study aimed to optimize irrigation strategies to enhance the yield and water use efficiency of drip-irrigated maize under membrane conditions in southern Xinjiang. We employed a weighing evapotranspiration meter to analyze changes in soil moisture, maize growth, water consumption, crop coefficients, and yield. The goal was to determine the optimal irrigation practices for maximizing yield and efficiency under these conditions. These findings provide valuable insights for guiding irrigation practices and maximizing production benefits in the context of limited water resources.

## 2. Results

### 2.1. Changes in the Soil Environment

**Figure 1:**
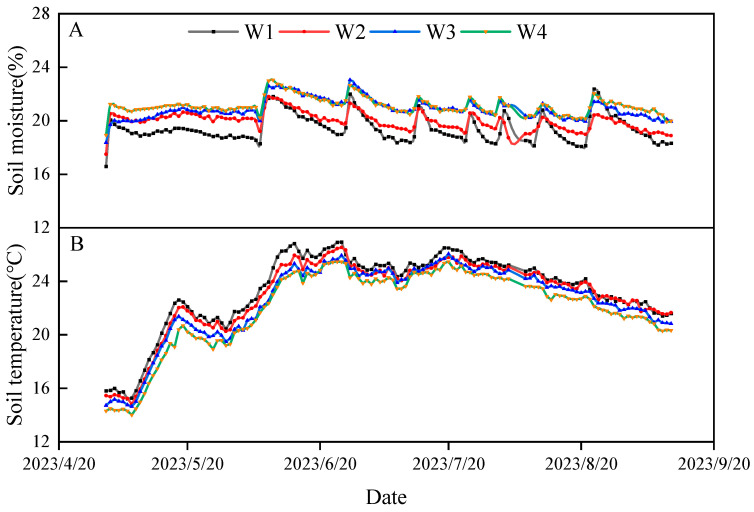
Changes in soil moisture and temperature during the maize growing period. (**A**) represents the fluctuation of soil moisture, while (**B**) represents the alteration of soil temperature.

The average soil moisture ranged from 16.59% to 23.05%. Soil moisture was relatively low before irrigation and significantly increased after irrigation. Soil moisture exhibited an increasing trend with the rise in irrigation volume, with the W2, W3, and W4 treatments showing increases of 2.86%, 7.51%, and 8.71%, respectively, compared to W1 (Figure 1A). The average soil temperature ranged from 14.01 °C to 26.95 °C, showing an initial increase followed by a decline as maize growth progressed. Compared to W1, soil temperature decreased by 1.67%, 3.48%, and 5.67%, respectively (Figure 1B).

### 2.2. Maize Growth

**Figure 2:**
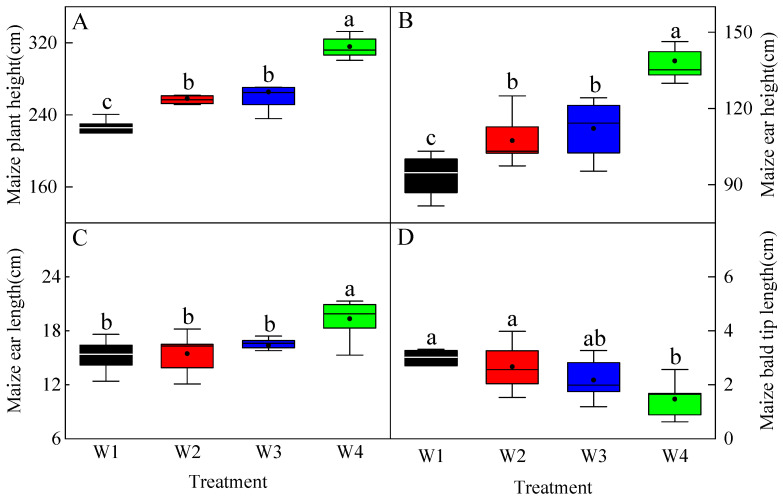
Effects of irrigation volume on maize plant height, ear height, ear length, and bald tip length. (**A**) represents the fluctuation of maize plant height, while (**B**) represents ear height. (**C**) represents ear height, and (**D**) represents bald tip length. Different lowercase letters indicate a significant difference in the mean value of different treatments at the probability level of 0.05 (*p* < 0.05), determined by a one-way analysis of variance (ANOVA) and Duncan’s post hoc test. The data are presented as means ± standard deviation (SD) calculated from nine maize samples.

The plant height, ear height, and ear length of maize all exhibited a significant increasing trend (*p* < 0.05) with the increase in irrigation volume. Compared to W1, the plant height in W2, W3, and W4 increased by 16.34%, 19.69%, and 42.38%, respectively (Figure 2A), while ear height increased by 16.85%, 22.10%, and 51.01%, respectively (Figure 2B). Similarly, ear length increased by 2.43%, 8.62%, and 28.13%, respectively (Figure 2C). The bald tip length of maize decreased with increasing irrigation, with W2, W3, and W4 treatments showing reductions of 6.62%, 23.85%, and 48.55%, respectively, compared to W1 (Figure 2D).

### 2.3. Soil Water Consumption and Crop Coefficient

**Figure 3:**
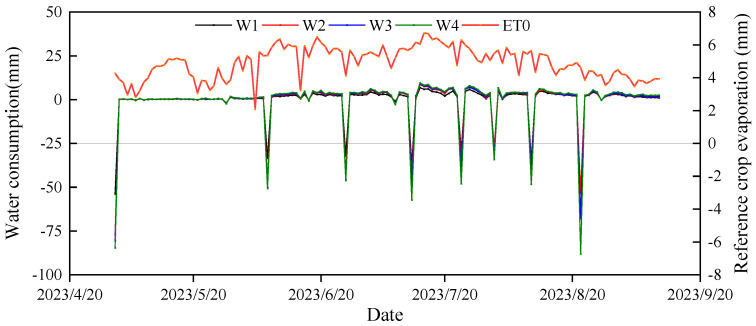
Soil water consumption during maize development.

Soil water consumption exhibited an “N” trend with the advancement of maize growth stages, with the maximum water consumption occurring during the tasseling and flowering stages, ranging from 88 mm to 132.16 mm. The average daily soil water consumption and crop coefficient initially increased and then decreased as the growth stages progressed, with the maize filling stage reaching its peak at 3.24–5.01 mm and 0.54–0.84 (Figure 3). Soil water consumption, average daily water consumption, and crop coefficient (Kc) all showed an increasing trend with increasing irrigation volume. Compared to W1, soil water consumption in the W2, W3, and W4 treatments increased by 24.56%, 33.57%, and 47.41%, respectively; average daily water consumption increased by 26.24%, 35.12%, and 49.45%, respectively; and crop coefficients increased by 13.43%, 21.16%, and 35.80%, respectively. Compared with ET, the total ET (W4KC) calculated by W1, W2 and W3 decreased by 119.70, 58.32 and 35.53, respectively (Table 1).

### 2.4. Maize Yield and Irrigation Water Use Efficiency

**Figure 4:**
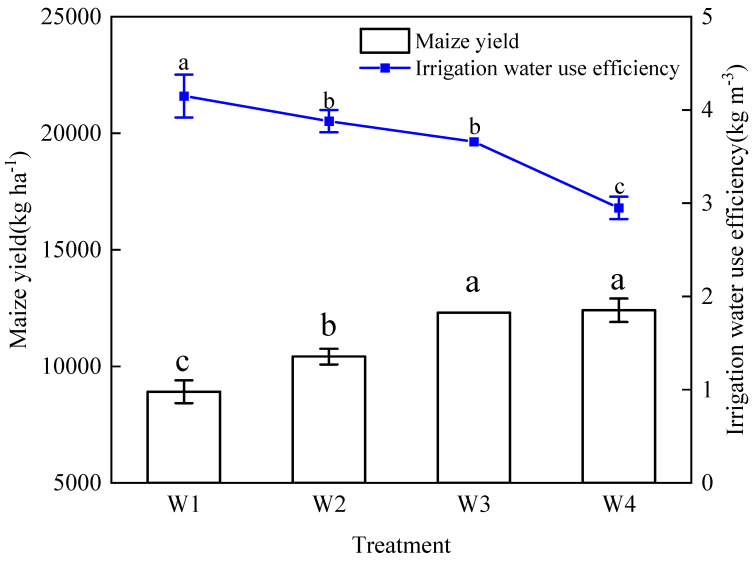
Impact of irrigation on maize yield and water use efficiency. Different lowercase letters indicate a significant difference in the mean value of different treatments at the probability level of 0.05 (*p* < 0.05), determined by one-way analysis of variance (ANOVA) and Duncan’s post hoc test. The data are presented as means ± standard deviation (SD) calculated from nine maize samples.

Irrigation significantly increased maize yield and reduced the irrigation water use coefficient (*p* < 0.05). Compared to W1, maize yield under W2, W3, and W4 treatments increased by 16.96%, 38.08%, and 39.24%, respectively, while the irrigation water use coefficient decreased by 6.57%, 11.85%, and 28.89%, respectively (Figure 4).

### 2.5. Complex Evaluation Based on TOPSIS

The comprehensive evaluation of TOPSIS based on maize yield, water consumption ET, and irrigation water use efficiency is shown in Table 2. The weight of ET is 32.97%, the weight of yield is 35.37%, and the weight of irrigation water use efficiency is 31.66%. The maximum value of index weight is yield (35.37%), and the minimum value is water use irrigation efficiency (31.66%). The TOPSIS score and grade under different irrigation conditions were the best (ranked first) for the W3 treatment, with a comprehensive score of 0.73, followed by the W4 treatment at 0.59, followed by the W2 treatment at 0.56, and finally the W1 treatment at 0.41. The significant score of the W3 treatment was higher than that of other treatments, indicating that the best treatment was the irrigation amount W3.

## 3. Discussion

Drought is the result of an imbalance between evapotranspiration flux (water loss from evaporation and plant transpiration) and water intake from the soil [34]. The results of this study indicate that the soil water content increased in conjunction with the increase in irrigation water. The W2, W3, and W4 treatments exhibited an increase of 2.86%, 7.51%, and 8.71%, respectively, in comparison to W1. This suggests that the soil moisture experienced cyclic changes due to irrigation, rainfall, and evapotranspiration, with irrigation and rainfall serving to supplement soil moisture and evaporation consuming soil water [35]. Consequently, increasing the irrigation amount can compensate for the water lost through plant transpiration to a certain extent and enhance the soil water storage capacity within the root area [36], which is consistent with the results of the studies of Nxumalo et al. [37] and Brar et al. [38]. The soil water content initially increases and subsequently declines with the progression of the growth period, reaching a relatively low value during the corn filling period. This is due to the fact that the corn filling period entails a transition from vegetative growth to reproductive growth, necessitating the provision of water for both these growth stages simultaneously.

Soil temperature initially increased and then decreased as soil fertility progressed. This pattern was attributed to soil temperature being influenced by energy absorption or release in response to variations in solar radiation and air temperature [39]. The high transmittance of the film allowed most of the film area to receive solar energy, leading to surface soil warming [40]. Additionally, water droplets on the film and water vapor in the air below the film facilitated the absorption of long-wave radiation, contributing to soil warming through the greenhouse effect [41]. As the maize canopy matured during later growth stages, it influenced the heat absorbed by the soil from solar radiation, which in turn affected soil temperature. An observed decrease in soil temperature with increasing irrigation volume indicated that higher soil water content reduced soil temperature. This effect occurred because soil temperature changes were primarily influenced by the thermal properties of the soil, including specific heat capacity, thermal conductivity, and thermal diffusivity. The soil’s specific heat capacity, which is influenced by its moisture content, increased with rising moisture levels, leading to a slower rate of temperature increase [42].

Strategies designed to regulate crop evapotranspiration have the potential to positively impact crop water use efficiency, thereby enhancing sustainable crop production [43]. The most prevalent method for calculating crop evapotranspiration involves multiplying the reference evapotranspiration (ET_0_) by the crop coefficient (K_c_), a process commonly referred to as the FAO56 method [44]. An alternative approach is to directly measure crop evapotranspiration using a gravimetric evapotranspiration meter [45]. The findings of this study indicate that maize water consumption and average daily water consumption increased with higher irrigation volumes. This suggests that increased irrigation effectively enhances soil water storage, which provides the necessary conditions for soil evaporation and plant transpiration [46].

Moreover, higher irrigation volumes promote maize growth and increase the surface area of maize leaves. A larger leaf area allows for more sunlight absorption, which subsequently increases the amount of energy converted into evapotranspiration (ET) [47]. Thus, the total evapotranspiration amount increases. Understanding the variability and magnitude of crop coefficients (K_c_) is crucial for accurately determining crop evapotranspiration (ET) and optimizing irrigation scheduling [48]. Crop biophysical parameters, such as leaf area index, ground cover, and canopy cover, are directly correlated with crop coefficients and can be used to estimate single crop coefficients or base crop coefficients for various crops [49]. In this study, it was observed that the maize crop coefficient (K_c_) and evapotranspiration both exhibited an increasing trend with higher irrigation volumes. This was because the crop coefficient was directly proportional to evapotranspiration, following the determination of reference crop evapotranspiration (ET_0_) through meteorological data analysis [50,51].

Maize proved to be highly sensitive to water deficit, which significantly impacted its growth and both its physical and chemical properties [52]. Water stress could severely impair maize growth and diminish its yield potential [53]. Conversely, over-irrigation was associated with yield loss, water wastage, soil salinization, and a decline in soil fertility [54]. The findings of this study revealed that maize plant height, ear height, and ear length all increased significantly with higher irrigation volumes, while bald tip length exhibited an opposite trend. These results suggested that a favorable soil hydrothermal environment promoted maize nutrient growth, with suitable soil moisture serving as a crucial resource for photosynthesis. Additionally, optimal soil temperature was found to enhance maize root respiration, which facilitated the transport of soil moisture and nutrients, thereby improving maize photosynthesis [55].

The results indicate that maize yield tends to increase with higher irrigation volumes. However, water use efficiency demonstrates the opposite trend, suggesting that excessive irrigation could lead to a substantial decrease in yield [56,57]. Proper irrigation was shown to support the accumulation of aboveground biomass, thereby improving both crop yield and water use efficiency [58]. Nonetheless, excessive irrigation could lead to fertilizer leaching, resulting in inefficient fertilizer uptake and reduced plant nutritional and reproductive growth, ultimately affecting crop yield [59]. The TOPSIS comprehensive evaluation confirmed this conclusion, indicating that while higher irrigation volumes led to increased maize yield, they also resulted in a notable reduction in irrigation water use efficiency. The data suggest that an irrigation volume of 3360 m^3^ ha^−1^ is a highly effective strategy for enhancing maize yield under membrane drip irrigation in the southern border area.

## 4. Materials and Methods

### 4.1. Overview of the Experiment Site

**Figure 5:**
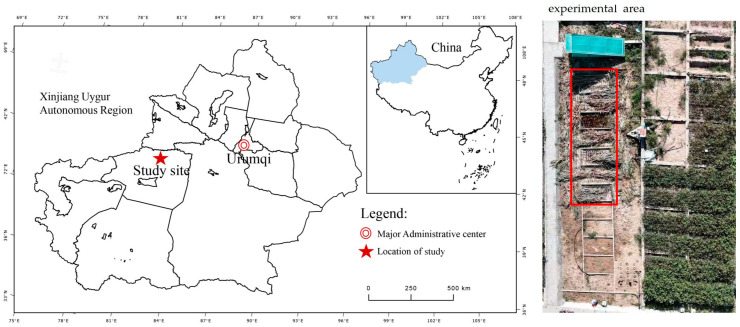
Location and layout of the study area.

The test area was situated at the base of the National Soil Quality Aksu Observation and Experiment Station in Baicheng County, Aksu Region, Xinjiang (81°54′22.6″, 41°47′37.2″; elevation 1232.1 m) (Figure 5). This region has a temperate continental arid climate, with an average annual temperature of 6.0 °C, an extreme maximum temperature of 38.3 °C, and an extreme minimum temperature of −28.0 °C. The frost-free period lasts between 133 and 163 days, and the mean annual sunshine duration is 2789.7 h. The average annual precipitation is 171.13 mm. The soil in the test area is classify as sandy loam, with a bulk density of 1.36 g cm⁻^3^. The soil exhibits medium fertility, with an organic matter content of 23.59 g kg^−1^, a total nitrogen concentration of 0.89 g kg^−1^, an alkaline hydrolyzable nitrogen level of 34.93 mg kg^−1^, an available phosphorus concentration of 21.81 mg kg^−1^, and an available potassium concentration of 120.92 mg kg^−1^. The pH value of the soil is 7.89, which is consistent with the average annual rainfall pH value [60].

### 4.2. Field Management and Experimental Design

In this experiment, a one-way completely randomized design was employed to establish four irrigation volumes of 2145 m^3^ ha^−1^ (W1), 2685 m^3^ ha^−1^ (W2), 3360 m^3^ ha^−1^ (W3), and 4200 m^3^ ha^−1^ (W4) for drip-irrigated maize under plastic mulch, based on the study conducted by Xiao et al. [61]. Fertilizers were applied via drip irrigation at rates of 241.5 kg ha^−1^ for nitrogen (N), 172.5 kg ha^−1^ for phosphorus (P), and 75 kg ha^−1^ for potassium (K). The remaining field management practices were consistent with local standards.

**Figure 6:**
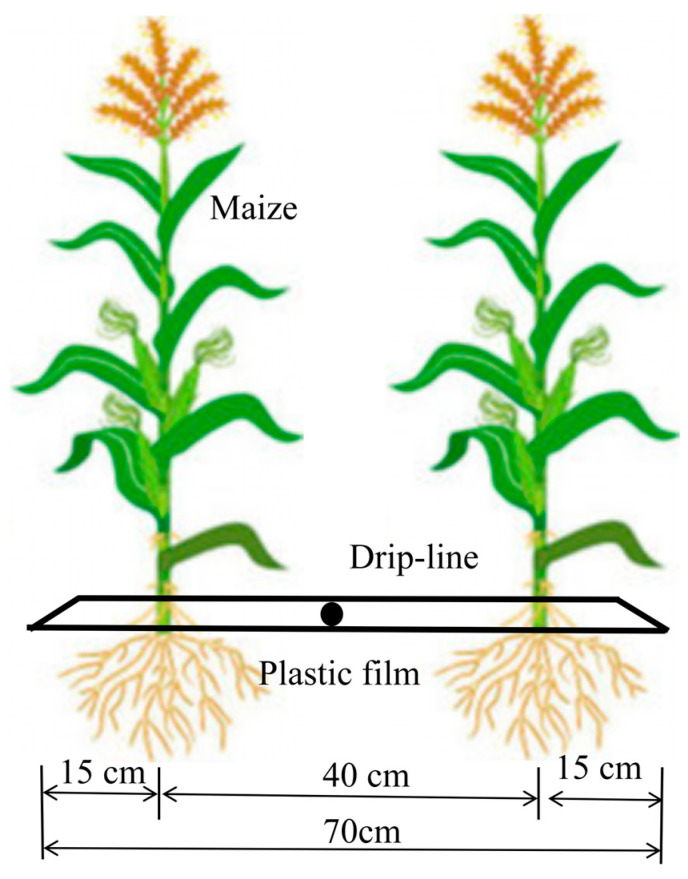
Diagram of maize planting arrangement and drip irrigation system.

Four treatments were implemented, with specifications of 3.3 m × 2 m × 2.3 m (length × width × depth), using a weighing soil evapotranspiration meter (XHZ-ZS201). The maize variety used in the experiment was Xinyu 31. It was sown on April 20, 2023, irrigated for the first time on May 1, and harvested on September 10. The planting was conducted with equal spacing between plants, with a row spacing of 40 cm and a plant spacing of 15 cm (Figure 6). An inlaid patch drip irrigation tape with a drip head spacing of 30 cm, a rated flow rate of 2.8 L h^−1^, and an operating pressure of 0.1 MPa was selected, according to the specifications outlined in the relevant literature. The source of irrigation water was groundwater, and the flow rate of the wells was sufficient to meet the required volume of water for irrigation purposes.

### 4.3. Measurement Items and Methods

#### 4.3.1. Soil Moisture and Temperature

An evapotranspiration meter (PH-30, Produced by Beijing Tanghua Technology Co., Ltd. Beijing, China) was installed with a soil water temperature sensor (recording frequency of once per hour) to monitor soil moisture and temperature at depths of 10 cm, 20 cm, 40 cm, 60 cm, 80 cm, 100 cm, and 130 cm during the reproductive period of maize.

#### 4.3.2. Maize Growth Indicators

During the maize harvest period, nine maize plants with uniform growth were randomly selected and labeled for each treatment. The maize plant height, ear height, ear length, and bald tip length were measured using a steel ruler with 1 mm graduations.

#### 4.3.3. Water Consumption and Crop Coefficient

**Figure 7:**
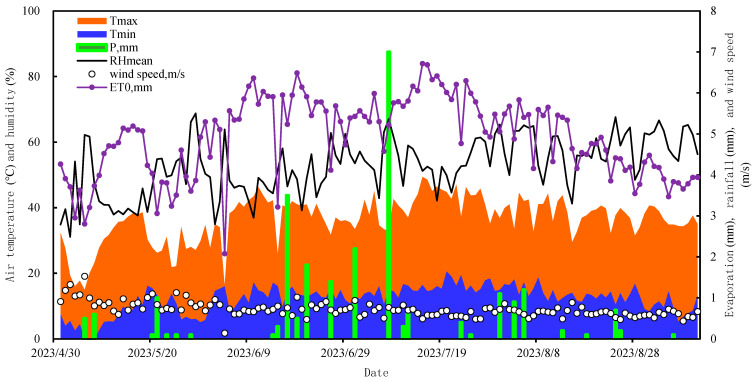
The 2023 meteorological data for the study area.

The calculation of crop water requirements (water consumption) for various irrigation activities was achieved using methods based on crop coefficients [62]. As outlined by Allen et al. [44], multiplying the reference evapotranspiration (ET_0_) by the crop coefficient (K_c_) provides the crop water requirement. K_c_ varies depending on crop type, phenological stage, and location. In this study, maize water consumption (ET) was measured using a lysimeter, and parameters such as daily maximum temperature (T_max_), daily minimum temperature (T_min_), average relative humidity (RH_mean_), wind speed, and precipitation (P) were obtained from a meteorological station (Figure 7). Maize K_c_ was calculated using Equation (1), and ET0 was calculated according to Equation (2) [63].

(1) $$K_{c}=\frac{ET}{ET_{0}}$$

(2) $$ET_{0}=\frac{0.408\times \Delta \times \left(R_{n}-G\right)+\gamma \frac{900}{T+273}\mu_{2}(e_{s}-e_{a})}{\Delta +\gamma (1+0.34\mu_{2})}$$

where ET_0_ is the reference crop evapotranspiration (mm d^−1^); Rn is the net radiation [MJ (m^2^ d)^−1^]; G is the soil heat flux [MJ (m^2^ d)^−1^]; γ is the psychrometric constant (kPa °C^−1^); T is the daily average air temperature (°C); μ_2_ is the wind speed at a height of 2 m above the ground (m s^−1^); e_s_ is the saturated water vapor pressure (kPa); e_a_ is the actual water vapor pressure (kPa); and Δ is the slope of the vapor pressure curve at temperature T (kPa °C^−1^).

#### 4.3.4. Maize Yield and Irrigation Water Use Efficiency Indicators

Maize yield was determined by harvesting all maize cobs in the evapotranspiration chamber at the point of maturity. The irrigation water use efficiency (IWUE) was calculated based on the methodology proposed by Wang et al. [64], as outlined in Equation (3):

(3) $$IWUE=\frac{Y}{I}$$

where Y is the maize yield (kg ha^−1^) and I is the irrigation water applied to the maize during the reproductive period (m^3^ ha^−1^).

#### 4.3.5. TOPSIS Integrated Evaluation

To evaluate the optimal strategy for efficient irrigation, we employed a multi-objective optimization method, the Technique for Order of Preference by Similarity to Ideal Solution (TOPSIS). This method reflects the current status by measuring the distance from the optimal and worst solutions. The computational process is based on the study by Abdoul Kader et al. [65].

TOPSIS was calculated as follows:

(1) Establishment of the contribution matrix of the evaluation indices:
  X = (Xij)n × m(4)
(2) Calculation of the normalized matrix:
  (5) $$\bar{X}=\frac{X_{ij}}{\sqrt{\sum_{i=0}^{n}X_{ij}^{2}}}$$
(3) Calculation of the weighted normalized matrix:
  (6) $$V_{ij}=\bar{X_{ij}}\times W_{j}$$
(4) Calculation of the Euclidean distances:
  (7) $$d_{i}^{+}=\sqrt{\sum_{j}^{m}{\left(V_{ij}-V_{J}^{+}\right)}^{2}}$$
  (8) $$d_{i}^{-}=\sqrt{\sum_{j}^{m}{\left(V_{ij}-V_{J}^{-}\right)}^{2}}$$
(5) Calculation of the performance score of the treatments:
  (9) $$P_{i}=\frac{d_{i}^{-}}{d_{i}^{+}+d_{i}^{-}}$$
  where Xij is the value of the ith treatment and the jth indicator; n and m are the number of evaluation indicators and the number of treatments, respectively; Wj is the weight of each evaluation indicator; $V_{J}^{+}$ and $J_{J}^{-}$ are the positive and negative ideal solutions, respectively; and $d_{i}^{+}$ and $d_{i}^{-}$ are the distances between the positive and negative ideal solutions, respectively.

### 4.4. Statistical Analysis

Experimental data were managed using WPS version 12.1.0.17147 (Kingsoft Office Software Co., Ltd., Beijing, China). A single-factor analysis of variance (ANOVA) was performed using DPS version 16.05 (Hangzhou Ruifeng Information Technology Co., Ltd., Hangzhou, China), followed by Duncan’s post hoc test to determine the significance of differences (*p* < 0.05 was considered significant). All graphs were generated using Origin 2017.

## 5. Conclusions

This study demonstrated that increasing irrigation water volume significantly improved soil moisture content, water consumption, and crop coefficients. Additionally, maize plant height, ear height, ear length, and yield all showed significant increases, ranging from 2.43% to 28.13%. Conversely, soil temperature, maize bald tip length, and irrigation water use efficiency showed notable declines. The comprehensive evaluation using TOPSIS revealed that an irrigation volume of 3360 m^3^ ha^−1^ was an effective strategy for enhancing maize yield in southern Xinjiang under membrane drip irrigation. This strategy resulted in a 23.96% reduction in irrigation water usage compared to the 4200 m^3^ ha^−1^ irrigation volume, while improving irrigation water utilization efficiency and reducing yield by only 0.84%. The findings of this study provide a theoretical foundation for optimizing production benefits under limited water resources.

## Figures and Tables

**Table 1:** Effects of different irrigation treatment on ET during maize growth period (with Kc obtained from complete irrigation W4 as a reference).

Treatment	Index	Jointing Stage	Bellbottom Stage	Heading–Flowering Stage	Filling Stage	Maturity Stage
W1	ET	3.54	17.09	88.82	55.00	86.00
	ET0	88.50	106.81	188.98	101.85	156.36
	ET(W_4_K_C_)	6.20	28.84	132.29	85.56	117.27
	ETd	0.18	0.78	2.69	3.24	2.61
	Kc	0.04	0.16	0.47	0.54	0.55
W2	ET	4.72	21.85	114.24	73.59	97.54
	ET0	78.67	109.25	190.40	102.21	154.83
	ET(W_4_K_C_)	5.51	29.50	133.28	85.86	116.12
	ETd	0.24	0.99	3.46	4.33	2.96
	Kc	0.06	0.20	0.60	0.72	0.63
W3	ET	5.58	24.00	123.34	77.40	104.20
	ET0	79.71	109.09	189.75	101.84	155.52
	ET(W_4_K_C_)	5.58	29.45	132.83	85.55	116.64
	ETd	0.28	1.09	3.74	4.55	3.16
	Kc	0.07	0.22	0.65	0.76	0.67
W4	ET	6.36	28.64	132.16	85.24	116.78
	ET0	90.86	106.07	188.80	101.48	155.71
	ETd	0.32	1.30	4.00	5.01	3.54
	Kc	0.07	0.27	0.70	0.84	0.75

Note: ET, ETd, and Kc denote water consumption, average daily water consumption, and crop coefficient, respectively.

**Table 2:** TOPSIS analysis of maize yield, soil water consumption, and irrigation water use efficiency.

Treatment	Euclidean Distances		Comprehensive Score Index	TOPSIS Rank
	d+	d−		
W1	0.83	0.56	0.41	4
W2	0.45	0.59	0.56	3
W3	0.29	0.78	0.73	1
W4	0.56	0.83	0.59	2

## Data Availability

The original contributions presented in the study are included in the article; further inquiries can be directed to the corresponding authors.
